# Teaching

**Published:** 1865-11

**Authors:** Wm. H. Atkinson


### Selections.

### TEACHING.

BY WM. H. ATKINSON, M. D.

[Read before the Northern Ohio Dental Association, May 3, 1865.]

They who engage in new enterprises are particularly anxious to have the *eclat* of what they esteem a respectable beginning. Prestige is not to be lightly esteemed, but the mere manner of *sowing* should in no case take precedence of sedulous care as to the kind and quality of the *seed* we desire to place in favorable position for germination.

The faithful horticulturist carefully and fully studies the habit of germination, growth, flowering and fruitage, as well as the soil in which his plants delight, and thus he is enabled to apportion them to the proper places as to richness or lightness of soil, exposure to sun, shade, etc., in accordance with the intent for which they are cultivated.

Those whose habit is luxurious growth, require pinching in and restraining methods of training, while those of delicate and feeble growth demand the kindest nursing and timely protection from the vicissitudes of season and weather, that untimely chill or sudden heat exerts no baleful influence upon them, so sure to blight the hoped-for crop of fruit or flower. All this knowledge is necessary to enable him to fill but not overcrowd his ground with plants. In case he have not seed enough to sow all over the prepared face of the soil, he will be obliged to work the harder to keep down the weeds that spring up in the unoccupied portions, but each plant may then be farther from its fellow, allowing it more soil from which to draw its nourishment, and thus have opportunity to transcend in size and quality the standard of its kind, and so repay for the increased space it occupies. Slow growers, that are only in flower when the season ends, must be removed to the hot-house to fruit, or be cut to take secondary places to make up the ensemble of bouquets composed of rarer flowers.

Our purpose like that of the horticulturist, is to bring out the highest culture of which our germs are capable and our soil can afford. In a word, our purpose is to make A No. 1 dentists (or at least contribute toward that high consummation) of those who enter our classes, holding that a work worth doing at all is fit to be wrought out in its highest possibilities. Those who grow so much to leaf and limb as to preclude flowering and fruitage the first year, we shall endeavor to induce to make terminal and winter buds, to enable them to hibernate until the vernal breezes and genial showers may awake them to the work of growing flower and fruit.

All those plants which persist in growing only leaf and limb, we shall transfer to the field whose work is "the healing of the nations," and those who refuse to come through, but persistently remain in a vigorous effloresence, we must assign to their legitimate field of labor in general surgery. But those who put forth well-formed limbs, covered with fully-developed leaves, through whose tufts protrude fragrant flowers that do not fail to give place to ripened fruit, we hail with open arms and throbbing hearts as the legitimate results of well-directed culture in dental science and art.

The highest ideal medical school would matriculate only those who pass the departments of botany, chemistry, pharmacy, anatomy, and physiology, to which they would add theory and practice of medicine.

The highest ideal of a school in general surgery would matriculate only graduates from the ideal medical school.

And the highest dental school would matriculate only graduates from the ideal school of general surgery, to which they would add principles of dental science and art, special anatomy and physiology, operative dentistry, artificial dentures and appliances, and a well-digested course of instructions in metallurgy.

But as none of these high ideals are as yet practical, we must content ourselves with our best endeavor to contribute toward their future adoption by those who can only take the elevated stand because of the preparation made by their humble but earnest predecessors.

The meeting of this body is an occasion that affords opportunity to present if it does not imperatively demand a somewhat extended survey of the function of teaching in general and particular.

Standing, as we of this age do, midway between the (genesis) origin of all things and (apocalypse) the ultimate sumation of philosophy in the fullness of comple revealment, it becomes us to so balance the authority of rigid conservatism, of finited "*reality*" and the limitless freedom and indefiniteness of "*ideal* extensity," as to hold ourselves above the one and within range of the other, so that both may be made subservient to our attainment of knowledge, without which fruit of mental labor we have no basis upon which to demonstrate fact or philosophy.

Teaching properly signifies the communication of the products of the labor of mind to mind; hence the hiatus between teacher and learner should be made as little felt as possible, that repulsion may be small and attraction great, thus favoring the mutual interblending of mind with mind, informing the teacher of the exact need of his pupil, enabling him the better to make the discovery of the solution sought, through the unperceived medium of magnetic contact.

The rapidity of the movement through which it is necessary for the mind to pass to attain the tension requisite to the solution of the problem before it, depends upon the inherent structure and relationship of the organs whose function it is to produce that which we denominate mind; hence there can scarcely be two minds exactly alike in celerity and clearness of perception and definition. So our standard of quick or slow will be formed by our observations of the minds with which we have come in contact. And this very diversity of endowment and culture becomes one of the greatest hinderances in the way of the regular and equal progress of a class of pupils who are to take the same course of study and demonstration; the fast fret at the slow for the delays they cause them, and the slow fret at the fast for their fuming, unreasonable impatience, and satisfaction with jumped-at conclusions, confidently averrring that anything so rapidly acquired must, in the nature of things, be as rapidly forgotten, and hence worthless. There is but one way to remedy this evil, and that is to allow each pupil to advance as fast as he may.

The duty of the teacher is not finished when he shall have gone through the prescribed routine of his curriculum, regardless of how well or illy it may have been apprehended by those to whom it was delivered, which is evinced by the haste to get through the irksome hour and slip from the desk with clandestine clerity, that the more pleasant association of the club-room, with "bon vivant" occupants who live on other men's earnings, may in some degree compensate him for the "*sacrifices* he makes for his pupils." Nay, he is not a teacher fit for his place who regards faithful performance of his every labor to advance his class as *a sacrifice*, in any other sense than that noble devotion of his best powers to the camplete fulfillment of the mission his Heavenly Father has committed to his charge, in the execution of which he can alone count himself happy.

The true teacher heartily regrets that his hour has so soon fled, and mourns that the rigidity of the prescribed rules must take precedence of the outgushing desire to open up the whole treasure house of mental stores that now, by the attrition of the effort to disseminate them, literally clamor for freedom of pronouncement, while the asking aspirations of the class hold teacher and pupil in the favoring mental tension by which to communicate and receive is making it not a task but a delight to both! When none but "teachers" occupy "chairs," and none but "pupils" fill the "seats" of schools, we shall hear less of "the dryness of study," and the "sacrifices of teaching."

The greatest evil that has befallen the whole range of teaching, sacred or secular, has been that incubus "pecuniary compensation!" which, "while most have coveted," a few have nobly ignored with "Him of blessed memory" as their head and type, "going about doing good" to all "without respect of persons." And yet it has been the abuse rather than the inherent iniquity of the practice, that has laid it under the anathemas pronounced against it. And until that which constitutes the currency of a country becomes the symbol of a given amount of labor which it truly represents, we shall not be able to extricate ourselves from the inconveniences complained of in the past and present by other means than through our educational influences of all kinds and degree, making it so disreputable to get something for nothing, or that which amounts to the same thing, a loose promise to pay in a medium of a flunctuating character and value, that no one can have the hardihood to perpetuate the dishonorable swindle.

Whenever we propose to pay persons for what they know, without the requirement that they satisfactorily impart it to those who listen and pay, we shall ever be in the predicament of vicarage, forced to accept the cheapest ability that is within reach of the principal to employ, after the example of manufacturers who engage the lowest grade of skill in the employees who produce their wares, so as to enhance the profits of the establishment, accruing to the stockholders.

Such is the commercial, I had almost said gambling, spirit of this age, that it is the general practice among our "best members of society" to wink at if not openly advocate the practice, the exercise of which has entailed upon us the inferior wares and doubtful morals that lay at the base of the infamous structure of false education in science and morals. Such is the jealousy and rivalry between conservative and novel methods of teaching that neither can hold undisputed sway.

All rivalry has its origin in the folly of entertaining the possibility of any one being able to take the place of any other. In the nature of things, each individual or combination of persons can only produce that which inheres in the organic nature which constitutes it. In a word, the stream can rise no higher than its source; the germ can no more than evolve the type stamped upon it by the parent stock.

Ready speakers are enviously dubbed "verbose," by those who claim to understand, but confess they are unable to teach what they say they so surely know.

A little observation will convince us that he who really knows will find means to prove his claim in one way or another, and vindicate the legitimacy of his mission. If he have no call to teach, he will soon learn that fact upon making the effort.

Whether we should give our preference to old or new teachers, will depend entirely upon which fulfills the demand made upon them to the best advantage and advancement of the pupil. That the first man must have been taught of God, is a proposition so plain as to command the concurrence of all; but in what manner the communications were made, is not so universally agreed upon. If we can but ascertain just how any mind comes into possession of knowledge, we shall have means by which to explain the manner of all acquisition of mental wealth. To comprehend this, we have to contemplate freedom and necessity of MIND.

If asked for a clear definition of what freedom of action is, we reply that any act itself not determined by some other act is free. The infinite mind is free because it includes all possibility of action.

Man is so far free as he partakes of the nature of the infinite uncaused acts of God. And he is bound in so far as he partakes of the serial positings of the Infinite in planetary bodies, which are, from the necessity of plus and minus expressions of force, confined within the law of *movement* producing them.

To illustrate: the human mind is free to contemplate infinity at pleasure so long as it remains negative or passive; but the instant it attempts to project itself into states of positive existence, it is forced to take the regular positings of numbers, if you please, in exact accordance with arithmetic modes of movement, or it cannot demonstrate to its own understanding the inherent powers of the aggregations, or separations of numbers. Hence, if we would solve any proposition, we must consent to travel within the rule ourselves, until the series of perceptions necessary to the focalization of mental attention be complete in the solution of each successive step in the process which becomes in the end the final solution or answer to the whole.

Our preference then should be given to that teacher, be he young or old, conservative or transcendental, who best is acquainted with the mental processes through which he has passed to acquire whatever attainment he may possess.

The fact of knowing is the mere function of mental existence.

The function of teaching is the fact of dissemination of germs of knowledge in mental soil not yet cultured in the production of these plants.

As all forms of individual being must be mature and in possession of a plus quantity of that which constitutes them before they can multiply themselves by propagation of their kind, so, too, must every mental labor be brought to maturity or fruitage before it can be successfully propagated upon other mental stocks, improving or deteriorating the quality of the new variety above or below the standard of the parentage from which it sprang.

Much has been done in the past by empirical gathering of fragmentary knowledge or portions of science, but if we desire each epoch to be wiser than the past, and to do something toward hastening the great consummation of all fact into the one grand philosophy of ultimated knowledge, it behooves us not to stick too closely to the confined and fractional philosophies of the past, whose formularies partake so much of the deciduous methods of their pronouncement as to be principally made up of the mere means of leverage to force us out of our primal ignorance into their confined leading-strings. Neither should we ignore the little wheat that the great mass does contain, under the misapprehension that it is all chaff; for the formularies, like the chaff of wheat, have served a useful purpose in protecting the precious seed from the devouring elements until it had matured, so as to germinate and bring forth abundantly in the richer and more refined and thoroughly prepared soil in which it is proposed to sow it broadcast with the improved skill of later cultivators.

Not fearing then to set free the young and robust mind to run at a tangent from the point of its departure, at the risk of bisecting and transcending the staid formularies of the vauntedly exact methods of the past, let us complacently contemplate the fanciful attampts of vigorous youth to grasp at one magnificent effort the whole range of mental acquisition in the solution of the great problem of ideal and real existence, which spontaneous movement is itself both proof and prophecy of the ultimate accomplishment of the desire. A movement which is to cure the dissensions between past and present methods, by harmonizing fact and philosophy, must originate beyond the sphere of popular prejudice or scholastic bigotry, in that quiet circle of thinkers, observers, and scholars who prize truth, and seek her for her own sake with an ardor that knows no abatement, even in the midst of multifarious discouragements that obstruct their way. "The tactics and drill of this warfare are not to be learned amid the smoke of battle, by the mere tyros and bigots who are in such hot haste to practice them, but must be brought thither by those who have been schooled into philosophic tastes and habits."

"When this class of teachers shall have been duly inaugurated in all the schools of all the departments of human learning, science will then have triumphed over error, and art over nature. Reason will then have unfolded the riddle of the universe, from its genesis to its apocalypse; and that cosmical idea toward which the Creator has been moving through mighty periods of creation, from the primordial planetary germ, by means of successive strata, floræ, faunæ, and human races and nations, will at length stand forth revealed in the fullness of its life and glory.

"At the height we have now reached in our contemplations, how wide the horizon! How grand the prospect! As from a lone eminence of faith, where the whole past present and future of our race are spread out at one view, we look down upon that divine system of the world in which the end is known from the beginning.

"We see long ages roll onward ere it shall all be fulfilled, vast literatures and civilizations shed like forest leaves in its fulfilling, and unspeakable glories crowding thick and fast to its culmination, until, blinded by the vision, we almost wonder that mortal may gaze and live.

"But we will not doubt His fatherly goodness who, having shown unto His human children even the far-off stars in their destined courses and periods, will surely deign not less that they should scan the track of His earthly promises, and give them some Pisgah where they may lie down and die content, that other generatious shall enter into that for which they have toiled."

To a corps of teachers thus endowed, and thus enthused with the importance and prospect of their holy mission, labor is rest, and the most arduous toil is joy, in view of the great results sure to follow the unselfish exercise of the work they have to do.

The function of teaching, like every other function, is self-compensating, by the increase of power and facility which it acquires in the tried and demonstrated fields of its labors, for the conquering of the untried and extended propositions which lie before it.—*Dental Cosmos.*

